# Plasma treatment effects on destruction and recovery of *Porphyromonas gingivalis* biofilms

**DOI:** 10.1371/journal.pone.0274523

**Published:** 2022-09-14

**Authors:** Qing Hong, Hongmin Sun, Meng Chen, Shaoping Zhang, Qingsong Yu

**Affiliations:** 1 Department of Mechanical and Aerospace Engineering, University of Missouri, Columbia, MO, United States of America; 2 Department of Internal Medicine, University of Missouri, Columbia, MO, United States of America; 3 Nanova, Inc., Columbia, MO, United States of America; 4 Department of Periodontics, College of Dentistry, Iowa University, Iowa City, IA, United States of America; Kwangwoon University, REPUBLIC OF KOREA

## Abstract

The objective of this study was to investigate the treatment effects of non-thermal atmospheric gas plasmas (NTAP) on destruction and the recovery (or re-colonization) of *Porphyromonas gingivalis (P*. *gingivalis)* in biofilms. *P*. *gingivalis* is a well-known keystone periodontal pathogen strongly associated with periodontal diseases, especially periodontitis. *P*. *gingivalis* biofilms were formed on stainless steel coupons and treated for 1, 2, and 5 minutes by NTAP of pure argon gas and argon+oxygen gas mixture. MTT assay, colony forming unit (CFU) counting assay and confocal laser scanning microscopy (CLSM) were used to assess the destruction efficiency. In addition, the plasma treated biofilms were re-cultured in the medium supplemented with antibiotics and oxidative stress sources to determine the synergy of the NTAP with other antimicrobial agents. The results showed the plasma treatment could result in 2.7 log unit reduction in bacterial load. The recovered biofilm CFU with NTAP treatment combined with sub minimal inhibition concentration of amoxicillin was 0.33 log units less than the biofilm treated with amoxicillin alone. The recovered biofilm CFU in NTAP groups was about 2.0 log units less than that in the untreated controls under H_2_O_2_ treatment. There was approximately 1.0 log unit reduction of biofilm CFU in plasma treated biofilm compared with untreated control under paraquat treatment. The plasma treated biofilms exhibited less resistance to amoxicillin and greater susceptibility to hydrogen peroxide (H_2_O_2_) and paraquat, suggesting that NTAP may enhance biofilm susceptibility to host defense. These *in vitro* findings suggested that NTAP could be a novel and effective treatment method of oral biofilms that cause periodontal diseases.

## 1. Introduction

Periodontitis is a common oral disease, with a prevalence of 45 to 50% in the world’s population [1]. In US, 42% of adults 30 years or older have periodontitis [2]. It is an inflammatory process in periodontium initiated by dysbiotic plaque bacteria. This extremely common oral disease results in the destruction of periodontal connective tissue and resorption of alveolar bone. Approximately 8% of the US adult population has the severe form of this disease that, if left untreated, may lead to tooth loss and is also mechanistically associated with systemic complications [2]. Two major factors contribute to the pathogenesis of periodontitis [3]: first, dysbiotic periodontal pathogens can directly damage the periodontal tissues through the secretion of toxic products; second, the dysregulated host response to periodontal pathogens, which results in release of excess inflammatory mediators (pro-inflammatory cytokines and matrix metalloproteinases), is also involved in the pathology of periodontitis. Specific bacterial species and bacterial complexes occur more frequently in diseased sites, while other bacterial species are associated with periodontal health or stable periodontal lesions [4].

*P*. *gingivalis* is a well-studied keystone periodontal bacterium that is frequently involved in periodontitis [5,6]. Conventional mechanical debridement (i.e., scaling and root planing) can achieve a temporary reduction of *P*. *gingivalis* together with other pathogens colonized in the subgingival plaque [6,7]. However, plaque bacteria cannot be effectively removed from the majority of periodontal pockets by this mechanical therapy alone. Infections recurred in a significant number of patients in studies by Wasserman [8,9]. Antimicrobial agents may further suppress the periodontal plaque bacteria and increase the benefits of the conventional mechanical treatment. Numerous systemic and locally-delivered antimicrobial agents have been evaluated for the treatment of periodontitis with various degrees of success [3,10–14]. However, a lack of effectiveness of the antibiotics used may be due to development of drug-resistant strains or the dampened metabolic state of the biofilm microflora [15]. To overcome the challenges imposed by the emergence of antibiotic-resistant biofilm bacteria, alternative antimicrobial approaches need to be developed. As a promising alternative approach, plasma treatment using non-thermal atmospheric gas plasmas (NTAP) could effectively kill microbes in localized and topical infections [16–22].

Plasma treatment is an innovative treatment modality, with advantages over other antimicrobial methods currently in use. It has the characteristics of an ideal tool, including a high degree of efficacy, fast action, penetrability, lack of toxicity, compatibility with different materials, and cost-effectiveness [23]. NTAP produce a potent cocktail of highly reactive chemical species, including reactive oxygen species (ROS), such as O, O_2_^-^, and OH, and reactive nitrogen species (RNS), such as NO and NO_2_ [24]. These plasma species are known to exhibit strong oxidative properties and can induce signaling pathways in bacterial cells. For example, oxidation of lipids and proteins that constitute the cell membrane resulted in the loss of their functions, and bacterial cells were found to die in minutes or even seconds in such plasma-induced environment [25]. Lee’s group [26] reported that plasma species penetrated the cell membrane of the microorganisms, leading to cell death in subsequent chemical reactions. Lu and co-workers’ results demonstrated that plasma treatment could effectively kill *P*. *gingivalis* in biofilms [27] with no harm to oral mucosa of rabbits [19].

Lima et al [28] and Lee et al [29] also demonstrated the effectiveness of plasma disinfection against *P*. *gingivalis*. In addition, our previous study [30] showed that NTAP as an adjunct therapy further dampened inflammatory response as demonstrated by the decreased protein levels of IL-1β and TNF-α as well as an enhanced expression of anti-inflammatory marker IL-10 in rat gingival tissue. The reduced bone loss upon NTAP treatment in the rat periodontitis model was partially mediated through the increased microbial killing of periodontitis-associated bacteria including *P*. *gingivalis*, *Aggregatibacter actinomycetemcomitans* and *Tannerella forsythia*. Lee et al demonstrated that using NTAP to treat titanium dental implants could prevent bacterial adhesion and biofilm formation on the surface as another useful application for NTAP in clinical application [31]. However, plasma treatment was not able to completely kill *P*. *gingivalis* bacteria in the biofilms. Moreover, there are few studies of the bacteria regrowth pattern after the plasma treatment.

In this study, we applied NTAP to *P*. *gingivalis* biofilm to examine the antimicrobial effects of plasma treatment and the biofilm recovery after the plasma treatment. The objectives of this study are: 1). To illustrate the mechanism of action of the NTAP on effective destruction of oral biofilms that are pathogenic for periodontal diseases, 2). To investigate the recovery pattern of plasma treated *P*. *gingivalis* biofilms under the stress of antibiotics (amoxicillin) and reactive oxygen species (H_2_O_2_ and paraquat), simulating host defense against infections.

## 2. Materials and methods

### 2.1. Biofilm culture

*P*. *gingivalis* (ATCC 33277) was purchased from ATCC (Manassas, VA, USA), and cultured in an anaerobic jar (R685025, Thermo Scientific, Waltham, MA, USA) for biofilm formation, by following the manufacturer’s instruction. Briefly, 1 ml of 5×10^7^ CFU/ml *P*. *gingivalis* suspension was cultured with 1 cm × 1 cm 316L stainless steel coupons in a 24-well plate in an anaerobic incubator. The medium used was Tryptic Soy Broth (TSB) supplemented with Hemin and Vitamin K3, as described in the instructional manual from ATCC. Biofilms were developed for 3 days or 5 days. The medium was replaced at day 2 and day 4.

### 2.2. Plasma treatments

**Fig 1:**
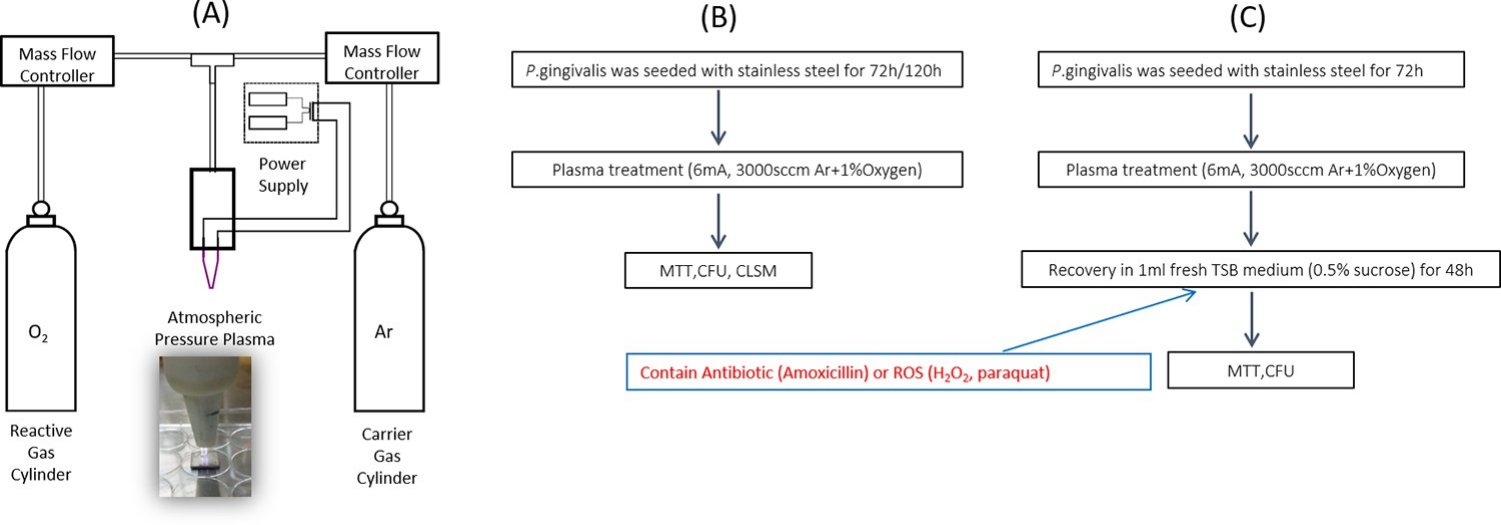
Experiment illustrations. A) sketch of the plasma brush, B) Experimental flow chart of the direct plasma destruction on biofilms, C) Experimental flow chart of the biofilm recovery after plasma treatment.

An NTAP brush used in this study was illustrated in Fig 1A. It was composed of a gas flow controller, a plasma generation chamber, and a direct current (DC) power supply to produce plasma. The details of the plasma brush have been reported previously [32,33]. Briefly, the argon and oxygen gas flow rates were controlled by a MKS mass flow controller (MKS Instrument Andover, MA, USA) and input into the plasma generation chamber containing two needle-shaped electrodes. A DC power supply (Spellman SL60) (Spellman High Voltage Electronics Corporation, Hauppauge, NY, USA) was used to ignite and sustain the plasma. The plasma brush was operated under 6 mA with feeding gas of either pure argon at 3000 standard cubic centimeter per minute (sccm) or argon/oxygen mixture of 3000 sccm argon + 30 sccm oxygen. These parameters had been optimized to achieve stable plasma with gas temperature of 37°C, which is the normal human body temperature.

Well-developed biofilms were rinsed 3 times with phosphate buffered saline (PBS) to remove the unattached bacteria. Coupons with biofilms on the surfaces were transferred onto the lid of a 24-well plate. An NTAP brush was applied to the biofilms by scanning for a preset time of 1, 2, and 5 minutes, respectively. The procedures were illustrated in Fig 1B.

### 2.3. MTT assay and CFU assay

MTT assay was used to assess the destruction efficiency of plasma against *P*. *gingivalis* biofilms. The plasma treated biofilm was stained with 0.5 ml MTT solution for 3 hours. MTT solution was removed and 0.4 ml of mixture of DMSO and alcohol (1:1) was added into the well to dissolve the stain. One hundred μl stain liquid of each sample was collected into a 96-well plate to measure optical density (OD) value at 575nm. The bacterial survival percentage was normalized with untreated control sample as 100%.

CFUs of the survival bacteria in the biofilms after plasma treatment were quantified. Each sample after treatment was transferred into a 5 ml centrifuge tube with 2 ml PBS. The tubes were then vortexed for 15 seconds and ultra-sonicated for 5 cycles to detach the bacteria from the coupons. The CFUs in the bacterial suspensions were counted by series dilution on agar plates. After 2 days of incubation, the CFUs were counted to assess the survived bacteria.

### 2.4. CLSM assay

Three-day old biofilms were stained with Live/Dead staining kit (L13152, Invitrogen, Waltham, MA, USA) by following the manufacturer’s instruction, and observed by a Carl Zeiss LSM 510 confocal laser scanning microscope (CLSM). Random locations of the biofilms were scanned with two channels of laser with an interval of 1 μm.

### 2.5. Biofilm recovery

Three-day biofilms were treated with plasma and gas blow. Afterwards, the treated biofilms were re-cultured in fresh TSB medium and incubated at 37˚C for 2 days. At the same time, biofilms that did not receive plasma treatment were cultured for 2 more days as controls. The recovered biofilms were assessed by MTT assay and CFU counting. The procedures were illustrated in Fig 1C.

### 2.6. Susceptibility to the antibiotic

During the recovery period, different concentrations of amoxicillin were added in the culture medium at the beginning of the recovery. After 2 days of recovery, MTT assay and CFU counting were performed to quantitatively assess the biofilms.

### 2.7. Susceptibility to oxidative stresses

Hydrogen peroxide and paraquat were used as the sources of oxidative stresses. During the recovery period, various concentrations of hydrogen peroxide and paraquat were added in the culture medium at the beginning of the recovery. After 2 days of recovery, MTT assay and CFU counting were performed to quantitatively assess the biofilms.

### 2.8. Statistical analysis

One-way analysis of variance (ANOVA) was performed on all data by using the SPSS statistics software (IBM, Armonk, NY). Tukey’s honestly significant difference post hoc test was performed to compare each group. If p < 0.05 is detected, these factors were considered to have a statistically significant effect at 95% confidence level.

## 3. Results

### 3.1. Plasma destruction on *P*. *gingivalis* biofilms

**Fig 2:**
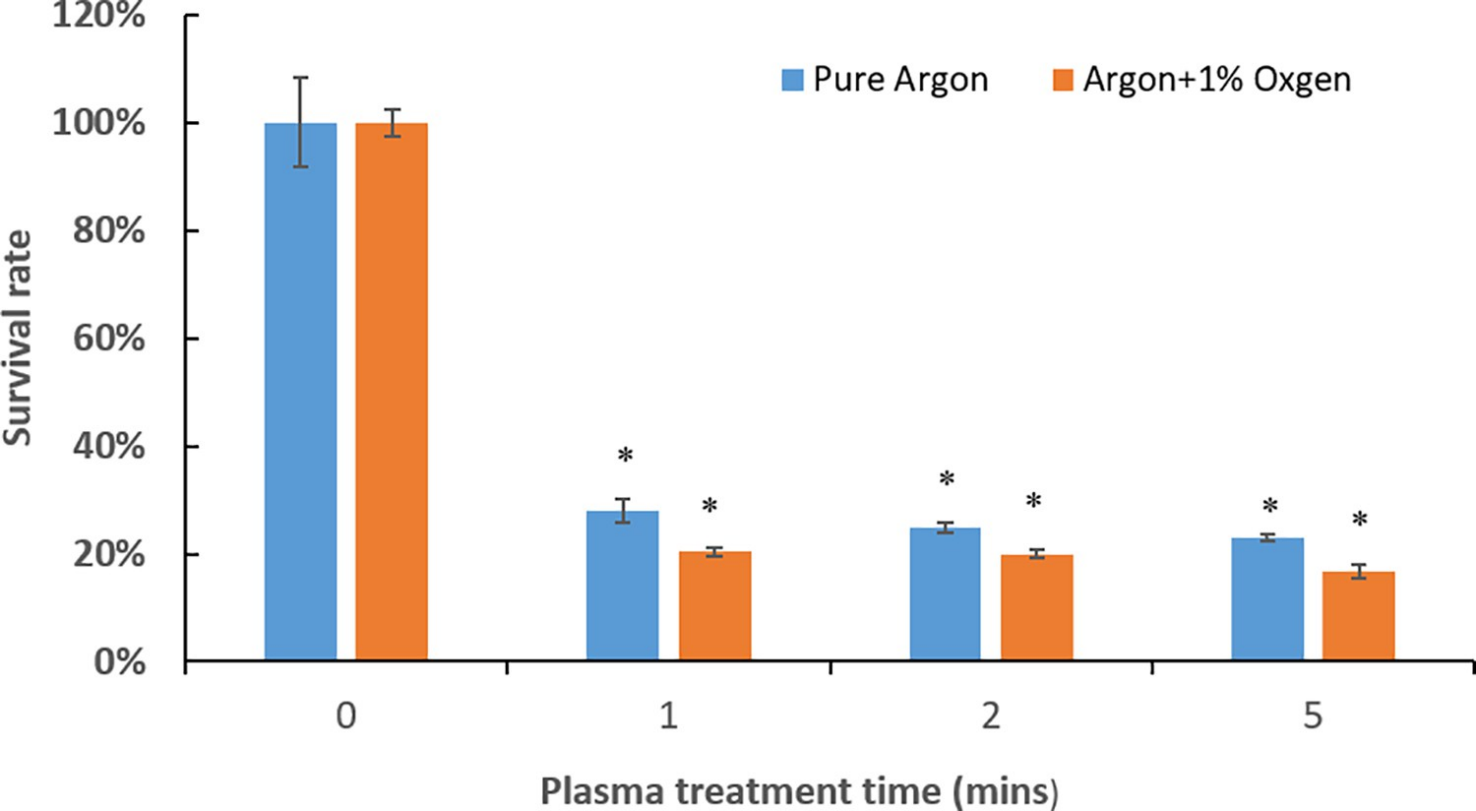
MTT assay results of the change of survival percentage of 3-day *P*. *gingivalis* biofilms with plasma treatment time. * denotes statistically significant difference compared with the control group with p < 0.05. Treatment time of 0 minutes represents the untreated control group.

**Fig 3:**
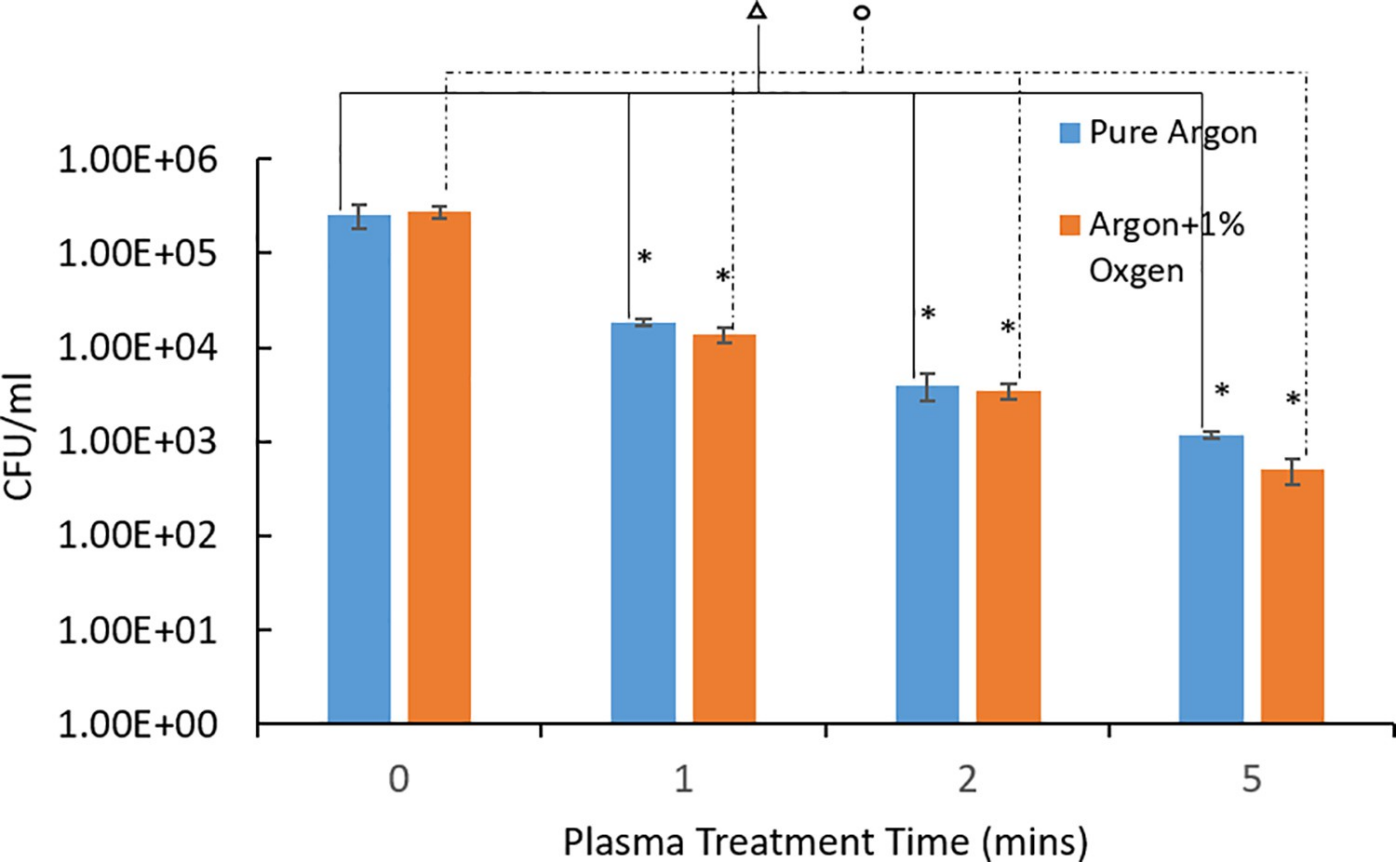
CFU counting results of plasma destruction of 3-day *P*. *gingivalis* biofilms after different plasma treatment times. Treatment time of 0 minutes represents the untreated control group. * denotes statistically significant difference compared with the control group with p<0.05 according to ANOVA Tukey test. denotes the statistically significant differences among different treatment times of pure argon plasma with p<0.05 according to ANOVA Tukey test. denotes the statistically significant differences among different treatment times of argon+1% oxygen plasma with p<0.05 according to ANOVA Tukey test.

NTAP’s effects on *P*. *gingivalis* biofilms on stainless steel were assessed. MTT assay was used to assess the destruction efficiency of plasma against *P*. *gingivalis* biofilms. Fig 2 presented the change of survival percentages of 3-day *P*. *gingivalis* biofilms with plasma treatment times. Plasma treatment exhibited high efficiency of destruction against 3-day *P*. *gingivalis* biofilms. Argon plasma treatment resulted in 76.97±1.21%, 75.09±1.80% and 71.95±4.41% bacterial load reduction with 1-, 2- and 5- min treatment time, respectively (p<0.05). Addition of 1 vol.% oxygen in the argon plasma improved the destruction efficiency, leading to 79.62±1.57%, 80.02±1.78% and 83.38±2.25% bacterial load reduction with 1-, 2- and 5-min treatment time, respectively (p<0.05). CFU counting assay (Fig 3) was consistent with the MTT results. A reduction of 2.3 log units of CFU (p<0.05) was achieved by the argon plasma treatment, while addition of 1 vol.% oxygen led to a further reduction of 0.4 log unit CFU (p<0.05).

**Fig 4:**
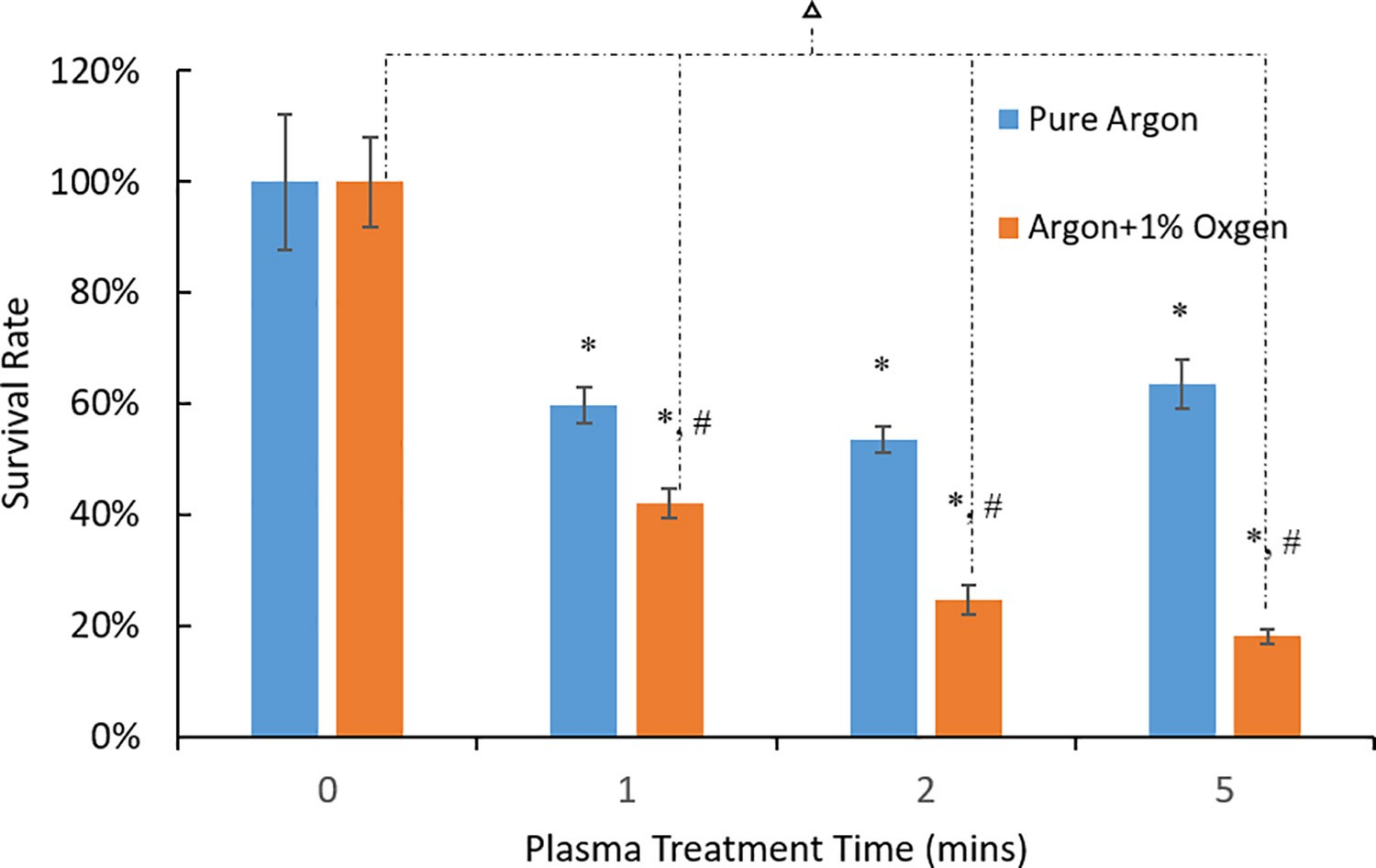
MTT assay results of plasma destruction of 5-day *P*. *gingivalis* biofilms. * denotes statistically significant difference compared with the control group with p < 0.05 according to ANOVA Tukey test. # denotes statistically significant difference between pure argon plasma and argon+1% oxygen plasma treatments with p < 0.05 according to ANOVA Tukey test. denotes the statistically significant differences among different treatment times of argon+1% oxygen plasma with p < 0.05 according to ANOVA Tukey test.

**Fig 5:**
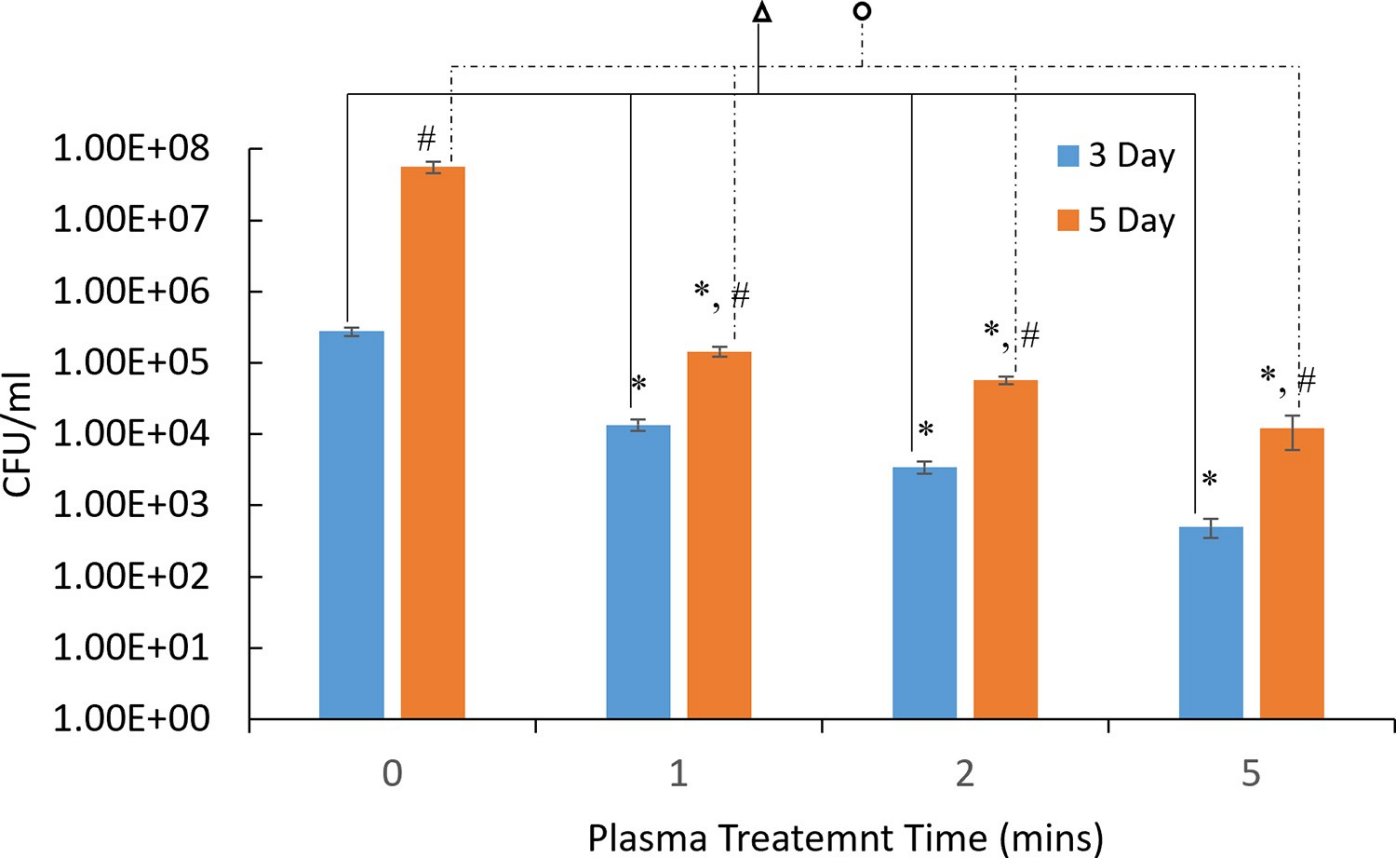
CFU counting assay results of argon+1% oxygen plasma destruction of 3-day and 5-day *P*. *gingivalis* biofilms. * denotes the statistically significant difference compared with the untreated control groups with p < 0.05. # denotes the statistically significant difference between 3-day and 5-day biofilms with p < 0.05 according to ANOVA Tukey test. denotes the statistically significant differences among different treatment times of 3-day biofilms with p<0.05 according to ANOVA Tukey test. denotes the statistically significant differences among different treatment times of 5-day biofilms with p < 0.05 according to ANOVA Tukey test.

Fig 4 shows MTT assay results of the plasma treatment effects on the 5-day *P*. *gingivalis* biofilms with different treatment times using pure argon plasmas and 1 vol.% oxygen addition into argon plasmas. Argon plasma treatment resulted in 40.24 ± 6.56%, 46.45 ± 4.78%, and 36.36 ± 8.91% bacterial load reduction with 1-, 2- and 5-min treatment time, respectively. Addition of 1 vol.% oxygen in argon plasmas further improved the disinfection, in agreement with results of treatment of 3-day old *P*. *gingivalis* biofilms. Addition of oxygen in the plasma reduced the bacterial load by 58.05±5.19%, 75.32±5.49% and 81.93±2.58% with 1-, 2- and 5-min treatment time, respectively. Fig 5 compared the plasma treatment against 3-day biofilms and 5-day biofilms. Plasma treatment with 1% oxygen addition resulted in less bacteria CFU (p<0.05) for 3-day biofilms (2.69 log units of CFU) than that for 5-day biofilms (4.07 log units of CFU). Therefore, only 3-day biofilms were used in the biofilm recovery studies.

**Fig 6:**
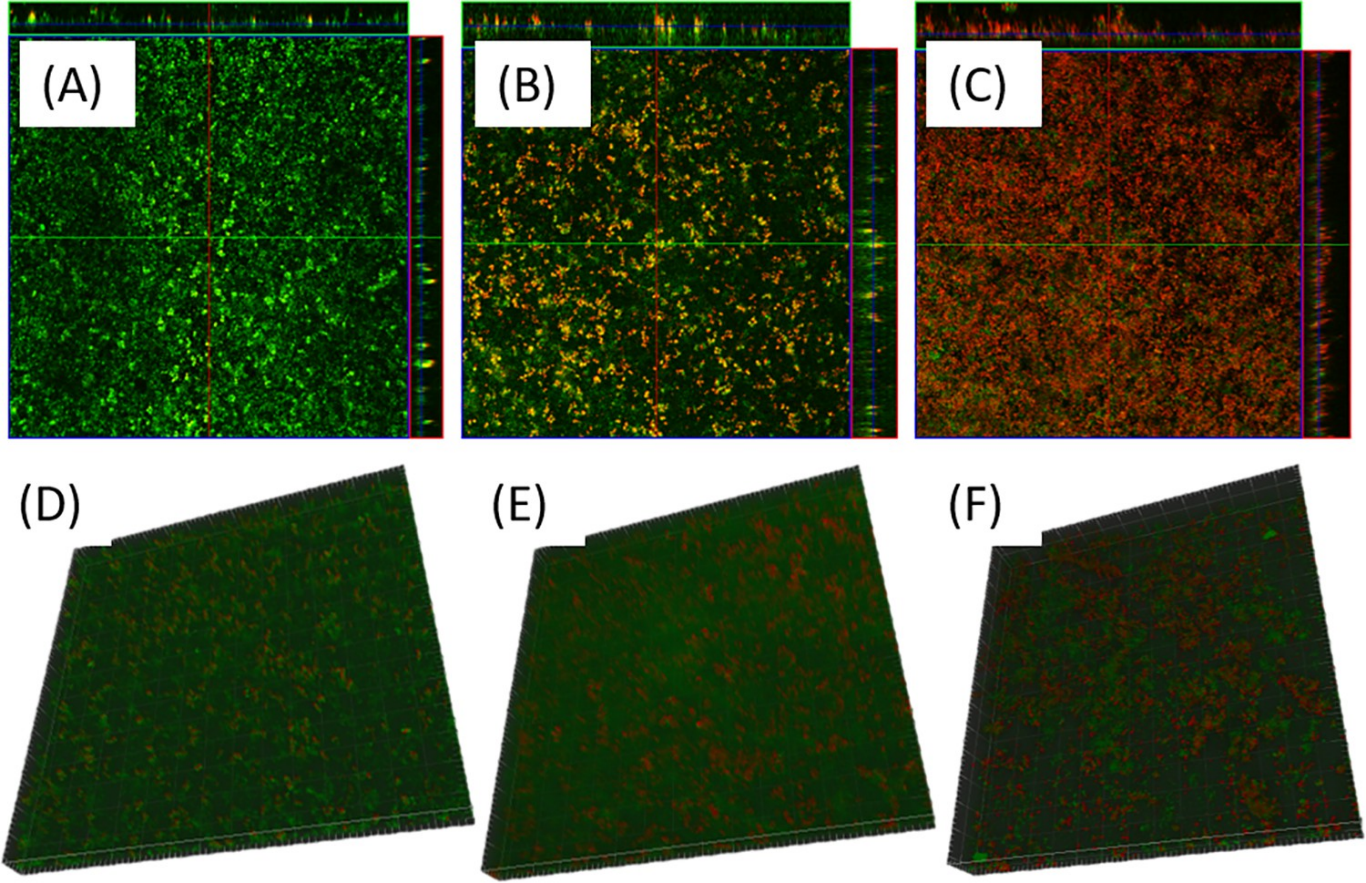
CLSM images of 3-day *P*. *gingivalis* biofilms. 2D images of: A) Untreated control, B) 1-min argon+1% oxygen plasma treatment, C) 5-min argon+1% oxygen plasma treatment; 3D images of: D) Untreated control, E) 1-min argon+1% oxygen plasma treatment, F) 5-min argon+1% oxygen plasma treatment.

The untreated and argon+1% oxygen plasma treated 3-day old *P*. *gingivalis* biofilms were examined using CLSM, with red color representing dead bacterial cells and green color representing living bacterial cells (Fig 6). As seen in the CLSM images, most of the cells in the untreated control group were alive. Dead bacterial cells appeared after 1-min plasma treatment, and a majority of the bacterial cells were dead after 5-min plasma treatment, indicating that plasma destruction of *P*. *gingivalis* biofilms was time dependent, being consistent with the results from MTT assay and CFU counting assay. Meanwhile, the plasma was able to penetrate into the biofilm structure to cause the biofilm destruction, as demonstrated in the 3D structure figures of the biofilms showing widespread distribution of dead cells (Fig 6E and 6F).

### 3.2. Recovery performance of plasma treated *P*. *gingivalis* biofilms

**Fig 7:**
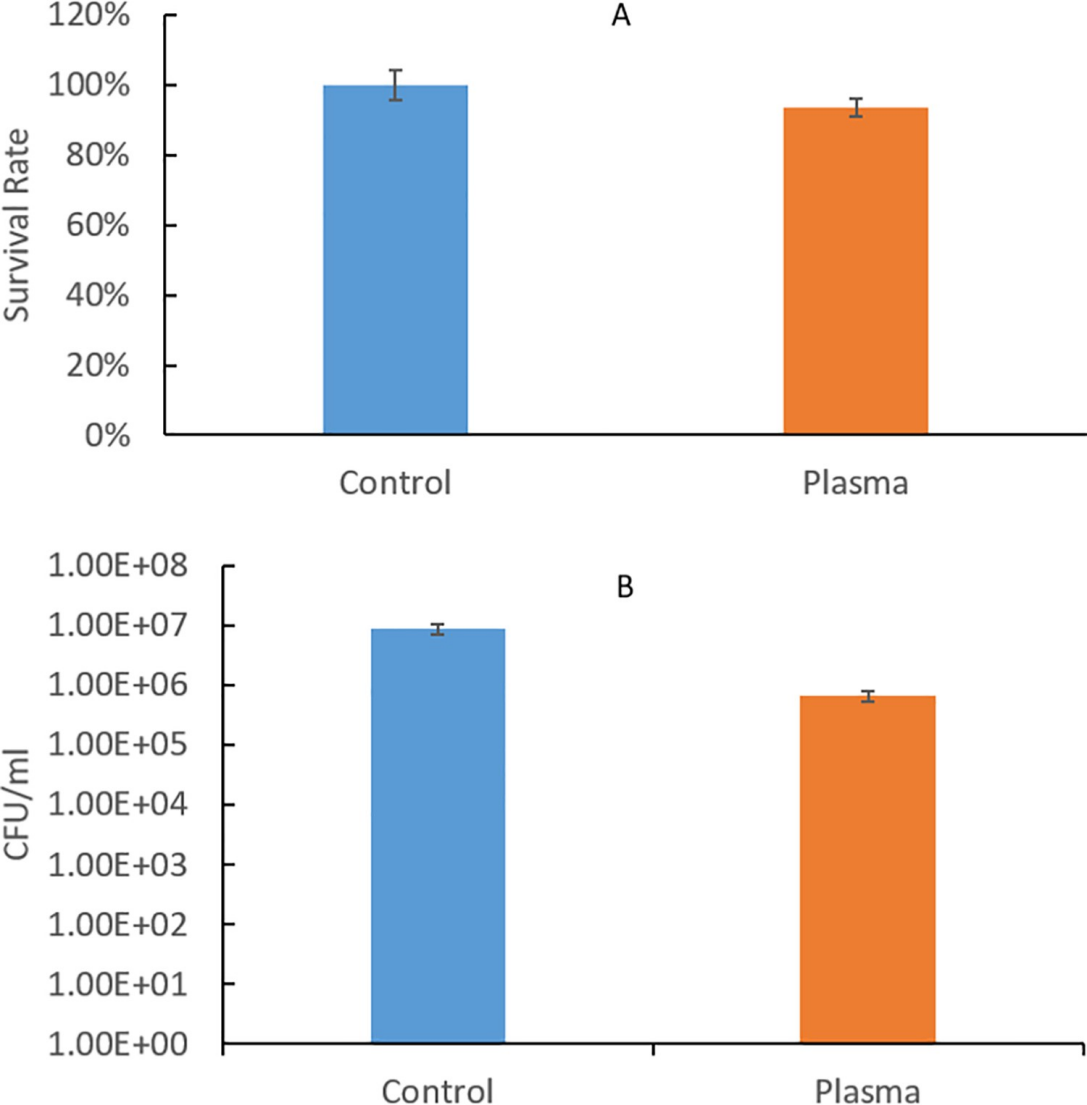
Bacterial recovery results from (A) MTT assay and (B) CFU counting assay for untreated and 1-min argon+1% oxygen plasma treated 3-day *P*. *gingivalis* biofilms.

The biofilm bacterial loads after 2 days of recovery from untreated and argon+1% oxygen plasma treated 3-day *P*. *gingivalis* biofilms were examined (Fig 7). There was certain but not significant reduction of bacterial load in plasma treated biofilms as compared to that in the untreated biofilms. MTT assay results showed that, after 2 days of recovery, the bacterial load in the plasma treated group was 6% less than that in the untreated control group. The biofilm CFU of the plasma treated group was 1.1 log units less than the CFU of the untreated control group. However, the difference between the control and plasma treated group was not statistically significant after 2 days of recovery.

**Fig 8:**
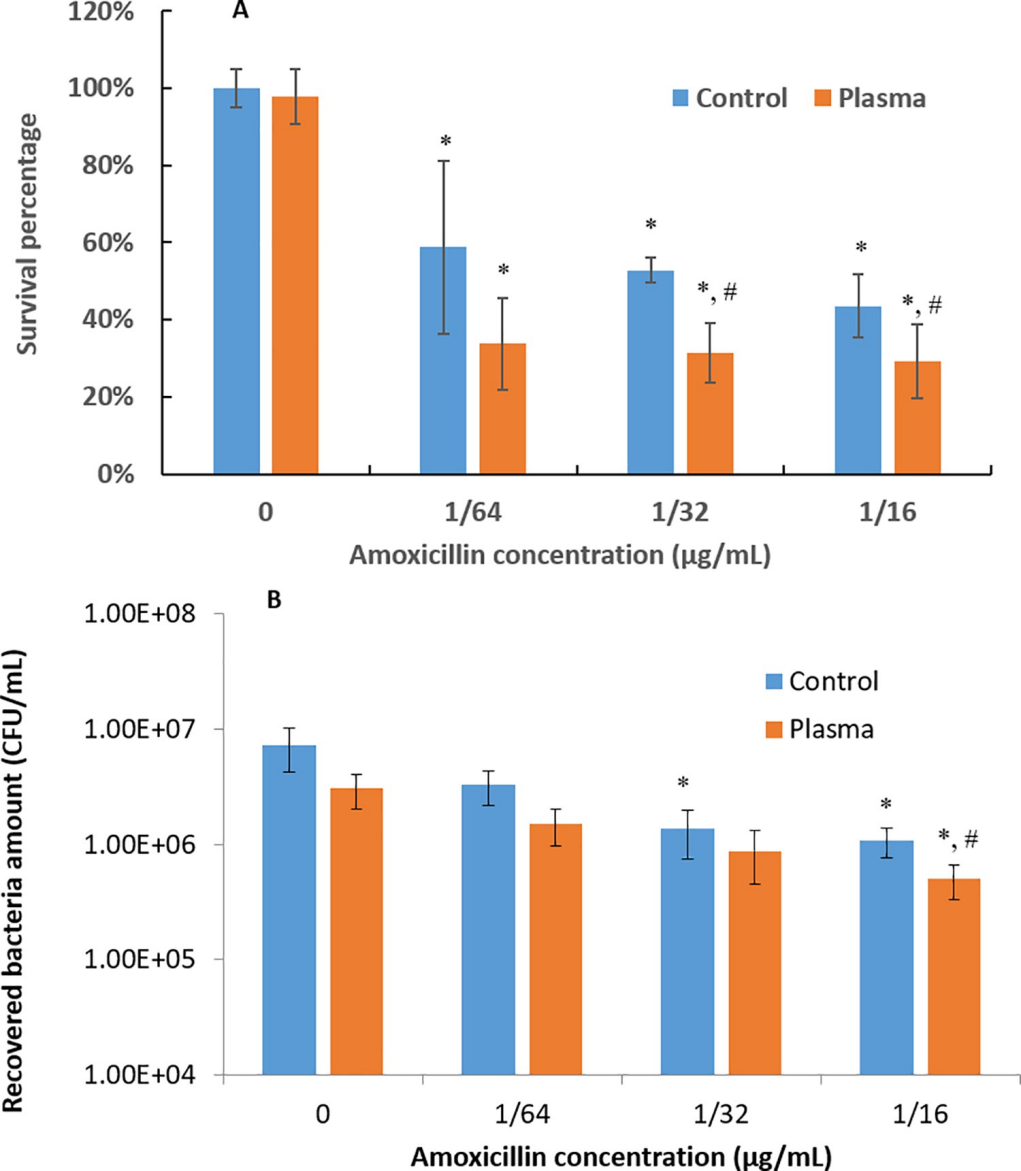
Antibiotic susceptibility results from (A) MTT assay and (B) CFU counting assay for untreated and 1-min argon+1% oxygen plasma treated 3-day *P*. *gingivalis* biofilms. * denotes the statistically significant difference (p < 0.05) compared with the control groups without amoxicillin application (0 μg/ml). # denotes the statistically significant difference (p < 0.05) between untreated controls and plasma treated groups.

Amoxicillin was applied as an antibiotic to prevent *P*. *gingivalis* biofilm recovery (Fig 8). Reported *in vitro* studies have demonstrated that minimal inhibitory concentration (MIC) of amoxicillin against planktonic *P*. *gingivalis* is around or less than 0.125 (1/8) μg/ml while bacteria in biofilms are more resistant to the antibiotic [14,34]. After 2 days of recovery under the stress of antibiotic, the bacterial survival percentage of argon+1% oxygen plasma treated *P*. *gingivalis* biofilms was 21.45% and 14.5% less (p<0.05) than the untreated biofilm controls for the groups with 1/32 and 1/16 μg/mL amoxicillin, respectively, in the recovery medium (Fig 8A). CFU counting results (Fig 8B) showed the same trend. Adding 1/16 μg/mL amoxicillin in the recovery medium, the biofilm CFU with argon+1% oxygen plasma treatment was 0.33 log units (p<0.05) less than the biofilm CFU without plasma treatment.

**Fig 9:**
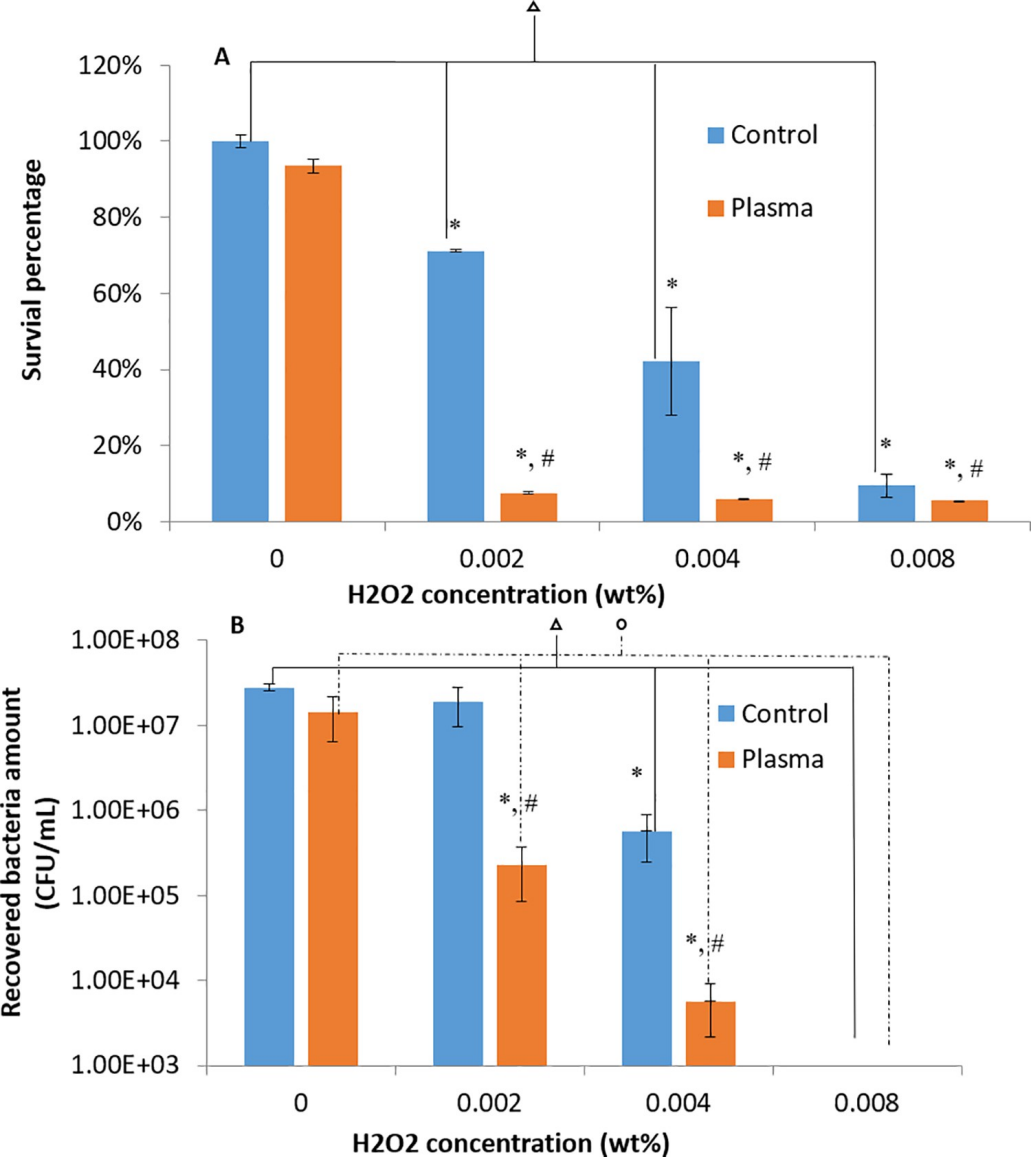
Biofilm recovery results under oxidative stress of H_2_O_2_ from (A) MTT assay and (B) CFU counting assay for untreated and 1-min argon+1% oxygen plasma treated 3-day *P*. *gingivalis* biofilms. * denote the statistically significant difference (p < 0.05) compared with the control groups without H_2_O_2_ treatment. # denotes the statisticall significant difference (p < 0.05) between untreated controls and plasma treated groups. denotes the statistically significant differences among different H_2_O_2_ doses for non-plasma treated biofilms with p < 0.05 according to ANOVA Tukey test. denotes the statistically significant differences among different H_2_O_2_ doses for plasma treated biofilms with p < 0.05 according to ANOVA Tukey test.

H_2_O_2_ was used as an oxidative stress inducer. The H_2_O_2_ responses by the untreated and plasma treated 3-day *P*. *gingivalis* biofilms were assessed (Fig 9). The H_2_O_2_ effects on *P*. *gingivalis* biofilms were concentration dependent. In comparison with untreated controls, plasma treated *P*. *gingivalis* biofilms were much more sensitive to H_2_O_2._ Even at a very low concentration of 0.002 wt% H_2_O_2_, 92.4±0.22% reduction in bacterial survival percentage was obtained. It should be pointed out that the similar inhibition effect for the untreated controls required a 4 time higher H_2_O_2_ concentration, i.e. 0.008 wt% H_2_O_2_, as shown in both the MTT assays (Fig 9A), and the CFU counting assay (Fig 9B). The recovered biofilm CFUs in the plasma treated groups were 1.91 and 2.00 log units less than (p<0.05) that in the untreated controls under H_2_O_2_ concentrations of 0.002 wt% and 0.004 wt%, respectively. When H_2_O_2_ concentration was higher than 0.004 wt%, all bacteria were dead in both plasma treated groups and the untreated controls.

**Fig 10:**
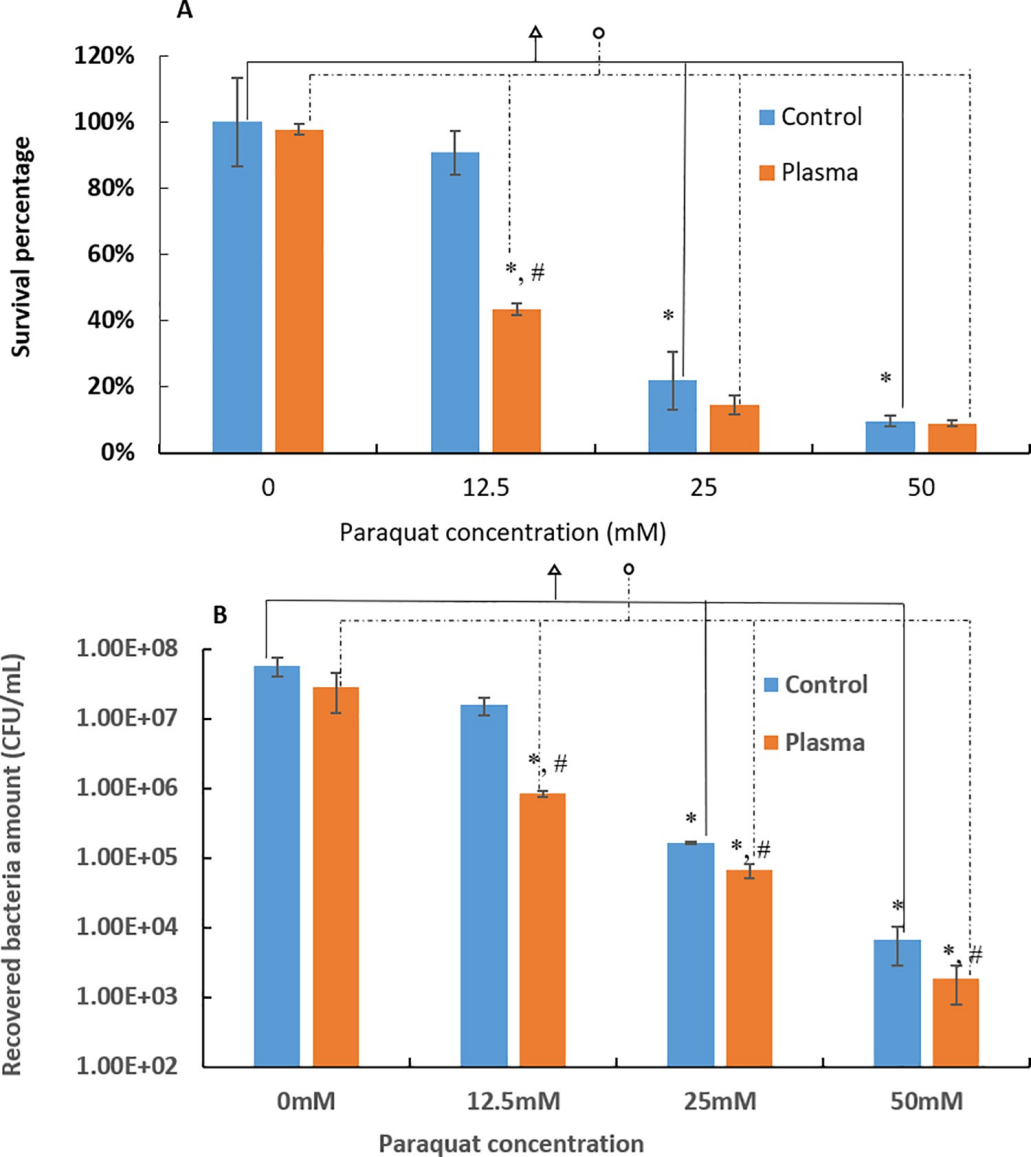
Biofilm recovery results under oxidative stress of paraquat from (A) MTT assay and (B) CFU counting assay for untreated and 1-min argon+1% oxygen plasma treated 3-day *P*. *gingivalis* biofilms. * denote the statistically significant difference (p < 0.05) compared with the control groups without paraquat treatment. # denotes the statistically significant difference (p < 0.05) between untreated controls and plasma treated groups. denotes the statistically significant differences among different paraquat doses for non-plasma treated biofilms with p < 0.05 according to ANOVA Tukey test. denotes the statistically significant differences among different paraquat doses for plasma treated biofilms with p < 0.05 according to ANOVA Tukey test.

Paraquat was used as another oxidative stress inducer. The paraquat responses by the untreated and plasma treated 3-day *P*. *gingivalis* biofilms were assessed (Fig 10). Plasma treated 3-day *P*. *gingivalis* biofilms were also more sensitive to paraquat treatment than the untreated control. The survival percentage of the plasma treated *P*. *gingivalis* biofilms was only 43.40±1.80%, which was significantly lower (p<0.05) than the 90.79±6.67% survival percentage observed with the untreated controls when treated with12.5 mM paraquat according to MTT assay (Fig 10A). Consistent results were also obtained with the CFU counting assay (Fig 10B). There was 1.5 log unit reduction (p<0.05) of biofilm CFU in plasma treated *P*. *gingivalis* biofilms while only 0.57 log unit reduction in the untreated control when treated with 12.5 mM paraquat (Fig 10B). Compared with the control groups without plasma treatments, the argon+1% oxygen plasma treatments were able to further reduce the bacterial load by 0.38 and 0.56 log units (p<0.05) when treated with 25 mM and 50 mM paraquat, respectively, in the recovery medium.

## 4. Discussion

Our recent research has shown that NTAP can rapidly and effectively disinfect not only planktonic bacteria of *Escherichia coli*, *Micrococcus luteus*, *Streptococcus mutans* and *Lactobacillus acidophilus* on various surfaces [20,35,36], but also bacteria in caries related biofilms [32,33] and fungus in candidiasis related biofilms as well [37]. The *in vitro* data collected in this study demonstrated that NTAP could be effective in destructing and reducing *P*. *gingivalis* biofilm reformation. MTT assay results showed that the bacterial load reduction in *P*. *gingivalis* biofilms was greater than 80% after plasma treatment by the argon+1% oxygen plasma for 5 minutes. CFU counting assay results also showed a 2.7 log unit reduction. These results indicated that NTAP could destroy the biofilms by killing the bacteria in *P*. *gingivalis* biofilms and therefore mitigate the pathogenicity of the biofilms.

In this study, 316L stainless steel, a medical grade of metallic alloy, was used as the surface for biofilm formation. 316L has been used in dental implants due to its good biocompatibility, excellent corrosion resistance, and high mechanical strength. Specifically, its applications in dentistry include: temporary crowns, arch wires, and brackets. Stainless steel coupons have been used by other research group for *P*. *gingivalis* biofilm growth [38]. Stainless steel was also utilized for studying adhesion of *S*. *aureus* to its surface for dental application [39]. Additional biomaterials used for making dental implants are titanium (Ti) and its alloy, Ti6Al4V. We are currently testing the plasma effect on biofilms formed on the titanium surface.

Plasma destruction efficiency of *P*. *gingivalis* biofilms depended on the biofilm structure and exposure time. Plasma treatment was less effective in microbial killing in 5-day *P*. *gingivalis* biofilms than that in 3-day biofilms, possibly because the biofilms were more mature after 5-day culture than after 3-day culture. There were more bacteria in the 5-day biofilms, and the mature biofilms provided stronger protection for the biofilm bacteria from the plasma treatment. The increase of plasma exposure time improved the killing of the biofilm bacteria, as it took time for plasma species to penetrate into the biofilm to destruct the bacteria in the biofilms.

In terms of plasma gas source, the addition of oxygen in argon plasma could improve the plasma destruction efficiencies on *P*. *gingivalis* biofilms. This can be attributed to the interaction of reactive oxygen species (ROS) in the plasmas with the bacteria. Production of more ROS with oxygen addition in the argon plasmas could lead to improvement of the destruction efficiency because ROS is one of the main plasma species causing disinfection. The ROS such as O^-^ and O_3_^-^ radicals in the plasmas can penetrate and destroy the microorganism structures through oxidation processes [40]. As reported by Lu et al. [41], the disinfection area enlarged significantly when the operating gas of He/H_2_ was replaced by He/O_2_.

Longer plasma treatment time was required to inactivate *P*. *gingivalis* biofilms, which could be due to the morphological structure of *P*. *gingivalis*. The ATCC 33277 strain of *P*. *gingivalis* used in the study is not capsulated standard strain. As a gram-negative anaerobic bacterium, *P*. *gingivalis* has an outer membrane, a peptidoglycan layer, and a cytoplasmic membrane [42,43]. The unique lipopolysaccharide structure of *P*. *gingivalis* might contribute its resistance to antimicrobial peptides [44]. Mahasneh *et al* also reported that *P*. *gingivalis* was less susceptible to plasma treatment than other bacteria [45].

Similar to the conventional mechanical treatment, it was very difficult to completely kill the bacteria in *P*. *gingivalis* biofilms with plasma treatment even though it exhibited high microbial killing efficiency against *P*. *gingivalis* biofilms. The recolonization of the plasma treated *P*. *gingivalis* biofilms was critical for clinical application of NTAP to thwart reoccurrence of the biofilm infections [46]. Bacteria regrowth will make eradication of biofilm infection difficult. In clinical practice, antimicrobial agents are sometimes used as an adjunct therapy to mechanical debridement. For example, amoxicillin is prescribed to patients with the molar-incisor pattern (formerly “aggressive periodontitis”) in conjunction with the scaling and root planing. Therefore, it is clinically relevant to investigate the recovery pattern of plasma treated *P*. *gingivalis* biofilms under the stress of antibiotics (amoxicillin) [34] and reactive oxygen species (H_2_O_2_ and paraquat), which simulate the host immune response in controlling oral biofilms [47].

Antibiotics, typically amoxicillin, are sometime prescribed as a combinatory antimicrobial therapy in conjunction with mechanical debridement or scaling and root planing under certain circumstances such as periodontitis with molar-incisor pattern or disease with refractory nature and resistant to treatment. Amoxicillin was tested in this study because it is the first line of the most frequently used antibiotic in clinic when antibiotics are regarded as necessary as an adjunct therapy. Previous studies on the minimal inhibitory concentration (MIC) of amoxicillin against 50% *P*. *gingivalis* clinical isolates in planktonic form is about 0.063 (1/16) μg/ml to 0.125 (1/8) μg/ml [14,34]. Different sub-MIC dosages of amoxicillin were tested in this study because such titrations would maximally expose the adjunctive effect of NTAP treatment in a titratable range. The results obtained in this study showed that plasma treated *P*. *gingivalis* biofilms were more susceptible to amoxicillin and their survival percentage was significantly decreased compared with that of the untreated biofilms. This finding indicates that the plasma treatment could increase the susceptibility of *P*. *gingivalis* biofilms to antibiotic treatment. In clinical practices, some patients with advanced periodontal diseases do not respond to conventional mechanical periodontal treatment, including surgical procedures, but need supplemental antibiotic treatments. Periodontal diseases are often associated with the presence of specific periodontal pathogens such as *P*. *gingivalis*, an exogenous pathogen that needs to be eradicated, if necessary by the administration of antibiotics [48]. The clinical outcome of antibiotic therapy is, however, not always satisfactory [49]. The most likely explanation of the treatment failure is that the subgingival bacteria present in biofilms are more resistant to antimicrobial treatment than planktonic cultures used for antimicrobial susceptibility testing [50,51]. The results obtained in this study indicate that, as a supplemental to the antibiotic therapies, plasma treatment has a significant benefit to improve clinical outcome.

In periodontal inflammation, polymorphonuclear leukocytes (PMN) served as the initial host defense against periodontal pathogens including *P*. *gingivalis*. After stimulation by bacterial antigens and during phagocytosis, PMN produces ROS such as hydrogen peroxide, single oxygen and hydroxyl radicals. ROS are the main elements for bactericidal activity of the host defense [52]. The antioxidant defense mechanisms of several bacteria were investigated by adding ROS-generating agents to the culture medium, which experimentally simulated the oxidative stress in host defense [47]. Hydrogen peroxide (H_2_O_2_) and paraquat are two widely used sources to provide ROS for *in vitro* experiments [53–58]. Hydrogen peroxide can produce singlet oxygen, superoxide radicals and hydroxy radical to damage the bacterial components such as enzymes, membrane constituents and DNA [59]. Paraquat can catalyze the formation of superoxide free radicals and cause toxic reactions such as peroxidation of polyunsaturated lipid, depolymerization of hyaluronic acid, inactivation of proteins and damage to DNA [60].

One of our previous studies [33] demonstrated plasma treated caries-related biofilms displayed significantly lower tolerance to the H_2_O_2_ and paraquat compared to untreated biofilms. In this study, these two ROS source reagents were used to assess the response of plasma treated *P*. *gingivalis* biofilms to oxidative stress. Our results demonstrated that plasma treated *P*. *gingivalis* biofilms exhibited significantly lower tolerance to H_2_O_2_ and paraquat, compared with untreated biofilms. The biofilms were more resistant to the attacking and killing by the host immune systems than planktonic bacteria, contributing to the enhanced virulence of biofilm infections [61,62]. Generation of ROS by PMN is one of the important steps to mediate the defense against bacterial pathogens. The results suggest that the plasma treated *P*. *gingivalis* biofilms could be better controlled by the host immune system than untreated biofilms. Although the *in vitro* generated oxidation environment by H_2_O_2_ and paraquat might not accurately recapitulate the *in vivo* generation of ROS by PMN in host defense system, our previous study showed that NTAP could not only reduce periodontitis-induced alveolar bone loss in rats but also reduce the periodontitis-associated bacteria burden, supporting our hypothesis that NTAP enhances host defense against periodontitis-associated bacterial infections [30].

## 5. Conclusion

In conclusion, plasma treatment using NTAP with both pure argon gas and its mixture with 1 vol.% oxygen demonstrated destruction efficacy on *P*. *gingivalis* biofilms. The addition of oxygen in argon plasmas further improved the plasma destruction efficiency. Plasma destruction efficiency on *P*. *gingivalis* biofilms also depended on the biofilm structure and treatment time. More importantly, the plasma treatment could significantly reduce the resistance of *P*. *gingivalis* biofilms to antibiotics and increase the susceptibility of the biofilms to oxidative stresses, suggesting plasma treatment could enhance host control of biofilm infection. Even though the plasma treatment could not completely prevent biofilm recovery, the recovery of *P*. *gingivalis* biofilms could be better controlled when the plasma treatment is applied in combination with other therapies. Future studies are still needed to assess NATP’s microbial killing efficiency of multi-species oral biofilms on other biomaterial substrates and the effect on inflammatory response in cell culture and the tissue repair after plasma treatment in animal periodontitis models. Plasma treatment using NTAP could have great potential to become an effective treatment method to disinfect and control pathogenic microorganisms in dental patients with periodontal diseases.
